# Senescence-associated secretory phenotype constructed detrimental and beneficial subtypes and prognostic index for prostate cancer patients undergoing radical prostatectomy

**DOI:** 10.1007/s12672-023-00777-1

**Published:** 2023-08-25

**Authors:** Dechao Feng, Jie Wang, Dengxiong Li, Ruicheng Wu, Wuran Wei, Chi Zhang

**Affiliations:** 1 Department of Urology, Institute of Urology, West China Hospital, Sichuan University, Chengdu, 610041 China; 2 Department of Rehabilitation, The Affiliated Hospital of Southwest Medical University, Luzhou, 646000 People’s Republic of China

**Keywords:** Prostate cancer, Biochemical recurrence, Senescence-associated secretory phenotype, Molecular subtypes, Meta-analysis

## Abstract

**Background:**

Cellular senescence is growing in popularity in cancer. A dual function is played by the senescence-associated secretory phenotype (SASP) that senescent cells produce in the development of pro-inflammatory niches, tissue regeneration or destruction, senescence propagation, and malignant transformation. In this study, we conducted thorough bioinformatic analysis and meta-analysis to discover detrimental and beneficial subtypes and prognostic index for prostate cancer (PCa) patients using the experimentally confirmed SASP genes.

**Methods:**

We identified differentially expressed and prognosis-related SASP genes and used them to construct two molecular subtypes and risk score. Another two external cohorts were used to confirm the prognostic effect of the above subtypes and risk score and meta-analysis was further conducted. Additionally, functional analysis, tumor stemness and heterogeneity and tumor microenvironment were also evaluated. We completed analyses using software R 3.6.3 and its suitable packages. Meta-analysis was performed by software Stata 14.0.

**Results:**

Through multivariate Cox regression analysis and consensus clustering analysis, we used VGF, IGFBP3 and ANG to establish detrimental and beneficial subtypes in the TCGA cohort, which was validated through other two independent cohorts. Meta-analysis showed that detrimental SASP group had significantly higher risk of biochemical recurrence (BCR) than beneficial SASP group (HR: 2.48). Moreover, we also constructed and validated risk score based on these genes to better guide clinical practice. DNA repair, MYC target, oxidative phosphorylation, proteasome and ribosome were highly enriched in detrimental SASP group. Detrimental SASP group had significantly higher levels of B cells, CD8+ T cells, homologous recombination deficiency, loss of heterozygosity, microsatellite instability, purity, tumor mutation burden, mRNAsi, differentially methylated probes and epigenetically regulated RNA expression than beneficial SASP group. The top mutation genes between detrimental and beneficial SASP groups were SPOP, FOXA1, KMT2C, APC, BSN, DNAH17, MYH6, EPPK1, ZNF536 and ZC3H13 with statistical significance.

**Conclusions:**

From perspective of SASP, we found detrimental and beneficial tumor subtypes which were closely associated with BCR-free survival for PCa patients, which might be important for the furture research in the field of PCa.

## Introduction

One of the most prevalent cancers affecting the male genitourinary system is prostate cancer (PCa) [1], of which there were 1,414,259 new cases worldwide in 2020, accounting for 7.3% of malignancies [2, 3]. Age is a risk factor of many diseases, such as periodontitis [4], neurological disorders [5], cardiovascular diseases [6], PCa [7, 8] and other cancers [9–16]. As population ages around the world, aging is putting more pressure on the healthcare system [17]. Nowadays, the primary treatments for the majority of patients with localised and locally progressed PCa were radical prostatectomy (RP) or radical radiation (RT) [18]. However, about 27%–53% [19–22] of patients undergoing RP and 10%–70% [23, 24] of patients RT will experience biochemical recurrence (BCR), which means the ascent of measurable PSA. BCR is regarded as a crucial node in the evolution of PCa and is linked to a higher likelihood of clinical recurrence, which raises the risks of metastasis and death [25]. As a result, timely BCR prediction and detection can accomplish the goal of early intervention and enhance patient prognosis.

Age is a well-known risk factor for PCa and cellular senscence is crucial to ageing and the emergence of disease [26]. The irreversible arrest of the cell cycle is considered as the typical feature of cellular senescence [27]. As a dynamic process, senescence cannot be studied as a static endpoint. Senescence research is not simple because it depends on a number of variables [28, 29]. Despite having a shared executive program, genotoxic stimuli cause random damage to cellular macromolecules, which leads to the possibility of cell-to-cell variation in the senescent phenotype [28]. In the situation of cellular senescence, cellular metabolism is significantly enhanced and senescence-associated secretory phenotype (SASP) refers to the ability of senescent cells to raise the quantity of cytokines, chemokines, matrix metalloproteinases (MMPs), and other proteins in the immediate surroundings [30–34]. SASP plays a significant role in numerous biological processes, including chronic inflammation, senescent cell clearance, stem cell activation, and tumour cell reprogramming, as a result of the huge synthesis of inflammatory cytokines, growth factors, and senescence-associated secretroy proteins [26, 33–36]. Early-stage senescent cells' secretomes may have anti-tumor effects, whereas late-stage senescent cells’. secretomes may promote inflammation and tumor growth [28]. On the one hand, senescent tumour cells can slow the growth of a tumour by attracting immune cells to the tumour microenvironment (TME) via SASP [37, 38]. On the other hand, the perennial persistence of SASP is considered to be detrimental. For example, MMPs are released by senescent fibroblasts, and they can facilitate tumour invasion and migration by rupturing the extracellular matrix (ECM) barrier and raising capillary permeability [39]. Additionally, they can encourage the release of additional growth factors and cytokines, such VEGF, which can facilitate angiogenesis and hence speed up the growth of tumours [40]. The ageing prostate can also contain signs of senescent cells in PCa, and SASP is connected to Pca [41]. The intricate mechanism of cellular senescence in PCa carcinogenesis and tumour progression, however, has not yet been fully understood. It is crucial to investigate potential prognostic markers for PCa from the standpoint of SASP and to get a thorough understanding of the mechanisms underlying them.

Based on three SASP-related genes, we created a risk score in our study and discovered detrimental and beneficial tumour subtypes, which may help guide clinical application going forward and predict the prognosis of PCa patients.

## Methods

### Data preparation and construction of prognostic risk subtypes and risk scores

**Fig. 1:**
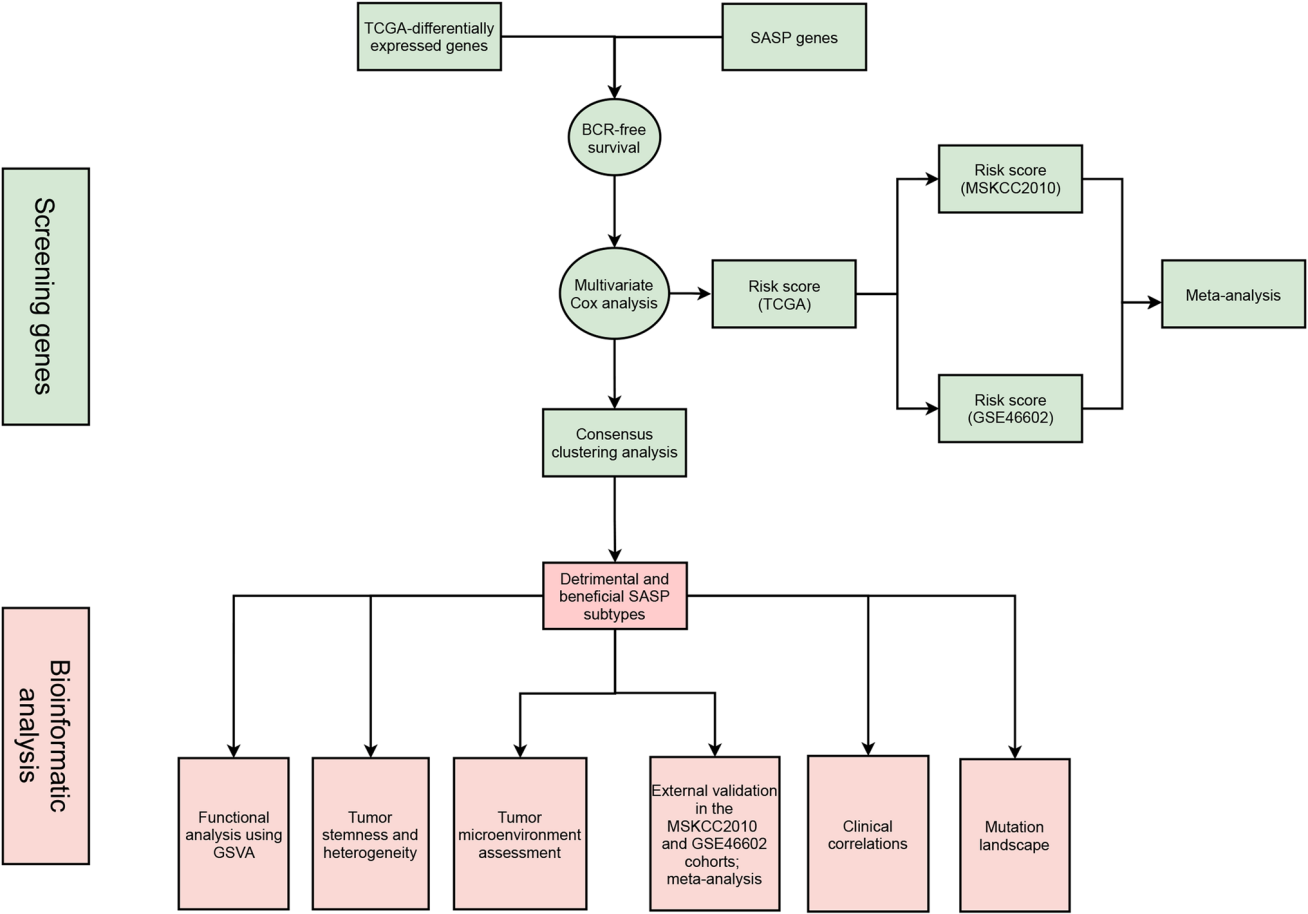
The workflow of this study. *SASP* Senescence-associated secretory phenotype

The workflow of our study is illustrated in Fig. 1. For the data preparation, we got the bulk-RNA seq matrix and clinical features in TCGA from our previous study [42]. 430 samples with complete BCR information were used. In addition, we downloaded two datasets as independent validation (GSE46602 [43] and MSKCC2010 [44]). We obtained a human SASP gene set from a previously published literature [45]. Using the R package “limma,” we then conducted a differential analysis of tumour tissue vs normal tissue within the TCGA cohort. The criteria for DEGs were llogFCl 0.4 and p.adj 0.05. Then we got the intersection between the DEGs and SASP genes. For the construction of prognostic risk subtypes and risk scores, we identified BCR-free survival-related genes using the above intersection results and univariate Cox proportional hazards regression analysis in TCGA cohort. Secondly, we used multivariate Cox proportional hazards regression analysis to determine the candidate genes. BCR-free survival-related genes were defined as those with a p value less than 0.05. Based on the BCR-free survival-related genes, we used consensus clustering analysis to divide the PCa patients in TCGA cohort into detrimental subtype and beneficial subtype. Meanwhile, we constructed a risk score based on these BCR-free survival-related genes and coefficient estimated through multivariate Cox analysis. Risk score = 0.202955141353009 * VGF + 0.292334174440823 * IGFBP3 − 0.241775198534725 * ANG. The differences of clinical features between detrimental subtype and beneficial subtype were analyzed. Two cohorts were used externally validated the prognostic values of risk score and SASP-related survival subtypes, including GSE46602 [43] and MSKCC2010 [44]. In addition, we merged the above results of risk score and SASP-related survival subtypes using meta-analysis through Stata 14.0 software.

### Mutation landscape and functional enrichment analysis

We downloaded RNA-sequencing profiles, genetic mutation and corresponding clinical information of PCa patients from TCGA database (https://portal.gdc.com). Based on R package “maftools”, we obtained and visualized the data of mutations. We also compared the differences of mutation frequency between these two SASP-related subtypes using chi-square test. In terms of functional analysis, gene set variation analysis (GSVA; GSVA is an open-source software package for R which forms part of the Bioconductor project and can be downloaded at http://www.bioconductor.org.) was performed using “h.all.v7.4.symbols.gmt” and “c2.cp.kegg.v7.4.symbols.gmt” from the molecular signatures database (MSigDB: http://www.broadinstitute.org/msigdb.) [46, 47]. The number of genes in the set ranged from 5 to 5000. The “wilcox.test” programme was then used to assess how each pathway differed between the two clusters. The log (fold change) was 0.4, and we defined statistical significance as a p-value of 0.05 and a false discovery rate of 0.05.

### Tumor stemness and heterogeneity analyses

As a cancer progresses, progenitor and stem-cell-like characteristics are acquired as a differentiated phenotype gradually disappears. Seven tumor stemness indices calculated by mRNA expression and methylation signature and OCLR algorithm [48] were obtained from a previous study [49]. For tumor stemness indexes, we compared differentially methylated probes-based stemness scores (DMPss), DNA methylation-based stemness scores (DNAss), enhancer elements/DNA methylation-based stemness scores (ENHss), epigenetically regulated DNA methylation-based stemness scores (EREG-METHss), epigenetically regulated RNA expression-based stemness scores (EREG.EXPss), RNA expression-based stemness scores (RNAss) and mRNAsi score between two subtypes. In addition to tumor stemness, tumor heterogeneity is another important feature of cancer. We obtained eight indicators about cancer from previous studies [50, 51]. For tumor heterogeneity, we compared homologous recombination deficiency (HRD) [50], loss of heterozygosity (LOH) [50], neoantigen (NEO) [50], tumor ploidy [50], tumor purity [50], mutant-allele tumor heterogeneity (MATH, R package maftools (version 2.8.05 inferHeterogeneity function), tumor mutation burden (TMB, R package maftools (version 2.8.05 tmb function) and microsatellite instability (MSI) [51] between two SASP-related survival subtypes. All these data could be obtained from our previous study [52].

### TME assessment

For the TME assessment, we used TIMER and ESTIMATE algorithms [53–55] to assess the overall TME and immune components. We also compared the differences of TME scores and some important immune checkpoints between these two SASP-related survival subtypes. Moreover, we calculated the Tumor Immune Dysfunction and Exclusion (TIDE) score to predict potential response of immune checkpoint blockade (ICB) therapy [56] and compared the differences between these two SASP-related survival subtypes. High TIDE score corresponds to poor efficacy of ICB. All the comparison of differences between these two SASP-related survival subtypes were based on the Wilcoxon rank sum test.

### Statistical analysis

All analyses were completed through software R 3.6.3 and its suitable packages. Meta-analysis was performed through Stata 14.0 software. D+L and I–V represent the meta-analysis results of random model and fixed model, respectively. We used Wilcoxon test to compare differences between groups for abnormal distribution. Survival analysis was conducted through log-rank test and presented as Kaplan–Meier curve. Statistical significance was set as two-sided p < 0.05. Significant marks were as follows: not significance (ns), p ≥ 0.05; *p < 0.05; **p < 0.01; ***p < 0.001.

## Results

### The identification of SASP-related survival subtypes and construction of risk score

**Fig. 2:**
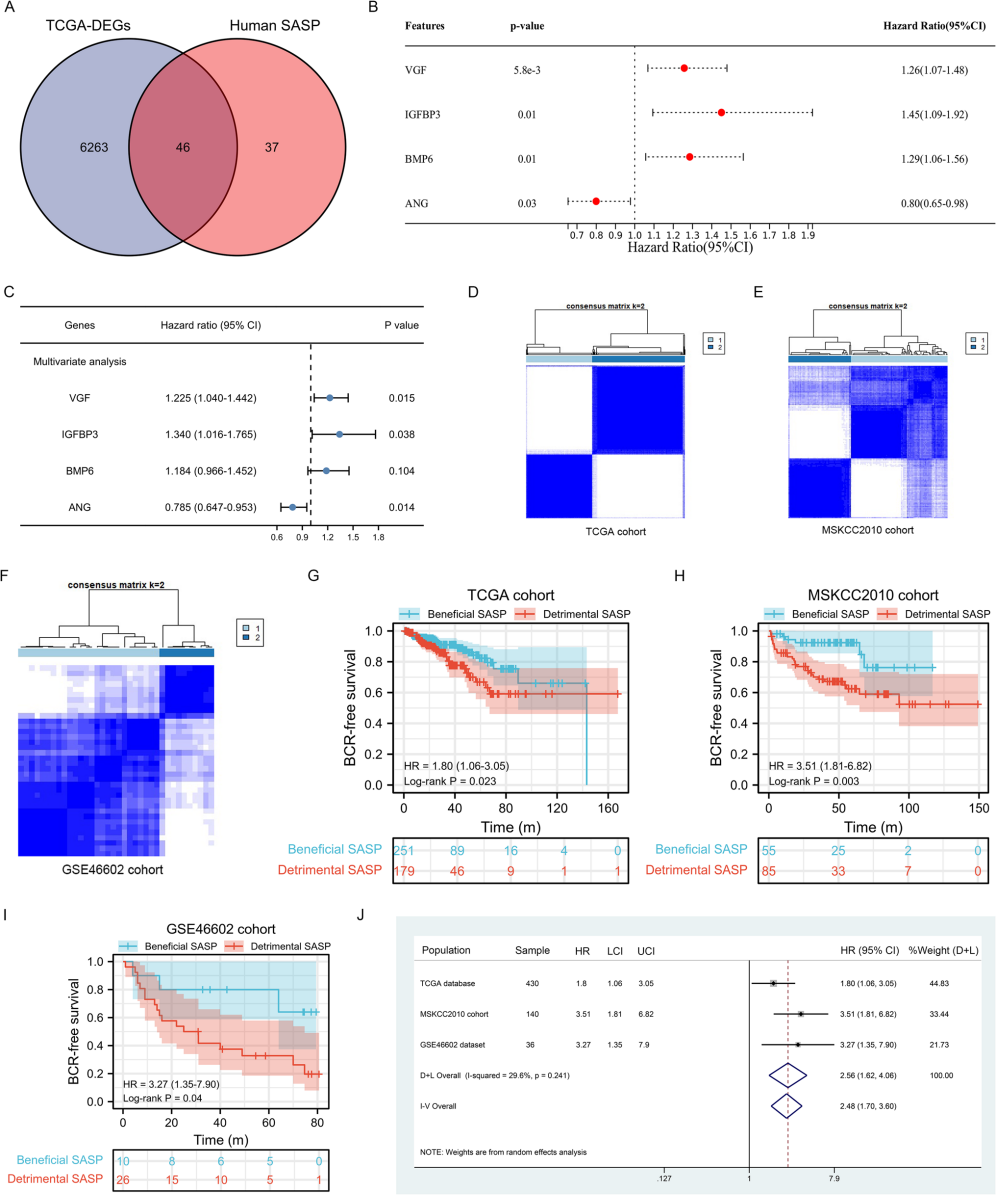
Identification of SASP subtypes in prostate cancer patients. **A** Venn diagram showing the intersection results of DEGs and human SASP genes; **B** univariate Cox regression analysis; **C** multivariate Cox regression analysis; **D**–**F** consensus clustering results of three cohorts; **G**–**I** survial curves showing the prognostic differences of the two subtypes in the three cohorts; **J** meta-analysis results of the above prognosis results. *SASP* Senescence-associated secretory phenotype, *DEGs* differentially expressed genes, *HR* hazard ration, *LCI* lower confidence interval, *UCI* upper confidence interval

**Fig. 3:**
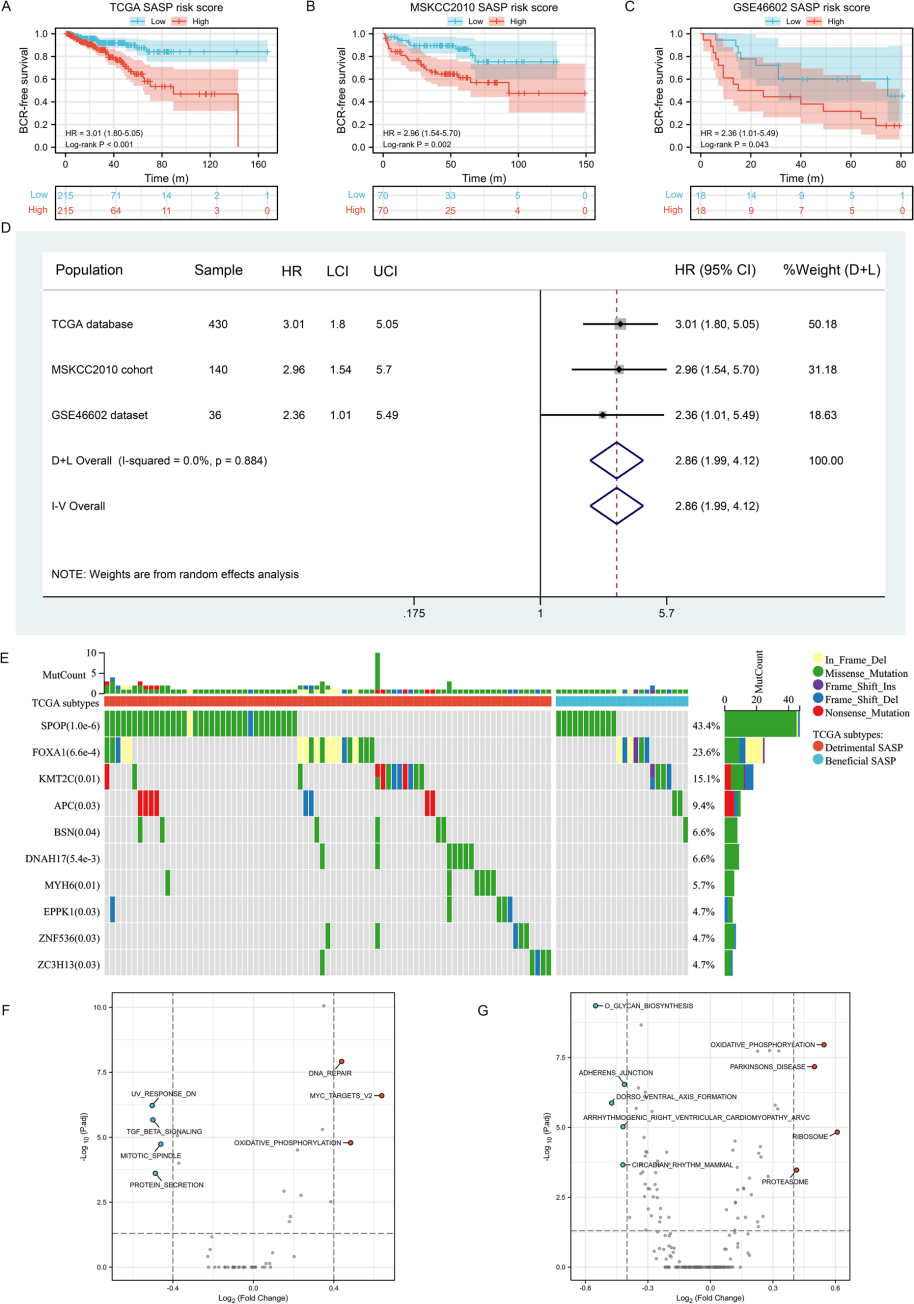
Construction of risk score, mutation analysis and functional analysis. **A**–**C** Survival curves showing the prognostic differences of the two risk groups in the three cohorts; **D** meta-analysis results of the above prognosis results; **E** waterfull plot showing the top ten mutation genes between the two subtypes; **F**, **G** functional analysis of the two subtypes. *SASP* Senescence-associated secretory phenotype, *HR* hazard ration, *LCI* lower confidence interval, *UCI* upper confidence interval

We conducted a differential analysis between 498 tumor samples and 52 normal samples in TCGA cohort and got 6309 DEGs (Fig. 2A). The DEGs were established as p.adj. 0.05 and llogFCl 0.4. Then after the intersection of DEGs and SASP genes, we got 46 genes for subsequent analysis (Fig. 2A). Using univariate Cox and multivariable Cox proportional hazards regression analysis, we finally obtained 3 candidate genes, including VGF, IGFBP3 and ANG (Fig. 2B, C). Based on these candidate genes, we subsequently used consensus clustering analysis to divide the PCa patients in TCGA cohort into detrimental subtype and beneficial subtype (Fig. 2D), which was confirmed using MSKCC2010 cohort (Fig. 2E) and GSE46602 cohort (Fig. 2F). The baseline comparison showed that patients in detrimental SASP subtype had higher Gleason score and positive residual tumor than beneficial SASP subtype (Table 1). Survival analysis in the TCGA cohort revealed that detrimental subtype had worse BCR-free survival than beneficial subtype (p = 0.023, HR [95%CI] 1.80 [1.06–3.05], Fig. 2G). Similar results were also observed in MSKCC2010 cohort (p = 0.003, HR [95%CI] 3.51 [1.81–6.82], Fig. 2H) and GSE46602 cohort (p = 0.04, HR [95%CI] 3.27 [1.35–7.90], Fig. 2I). Meta-analysis showed detrimental subtype had 2.48 times of risk of BCR than beneficial subtype (Fig. 2J). In addtion, we orchestrated a risk score based on these candicate genes and divided 430 PCa patients into high-risk group and low-risk group according to the median value of risk score. We found that high-risk group had worse BCR-free survival that low-risk group (p < 0.001, HR [95%CI] 3.01 [1.80–5.05], Fig. 3A). Similar results were also observed in GSE46602 cohort (p = 0.002, HR [95%CI] 2.96 [1.54–5.70], Fig. 3B) and MSKCC2010 cohort (p = 0.043, HR [95%CI] 2.36 [1.01–5.49], Fig. 3C). Meta-anlysis showed high-risk group had 2.86 times of risk of BCR than low-risk group (Fig. 3D).

**Table 1:** The clinical baseline of detrimental and beneficial SASP subtypes in prostate cancer in the TCGA database

Features	Detrimental SASP	Beneficial SASP	P value
Sample	179	251	
Age, median (IQR)	61 (56, 66)	61 (56, 66)	0.616
Gleason score, n (%)			0.006
6	9 (2.1%)	30 (7%)	
7	79 (18.4%)	127 (29.5%)	
8	25 (5.8%)	34 (7.9%)	
9	66 (15.3%)	60 (14%)	
T stage, n (%)			0.813
T2	63 (14.9%)	92 (21.7%)	
T3	111 (26.2%)	150 (35.4%)	
T4	4 (0.9%)	4 (0.9%)	
Race, n (%)			1.000
Asian	5 (1.2%)	6 (1.4%)	
Black or African American	21 (5%)	29 (7%)	
White	150 (36.1%)	205 (49.3%)	
N stage, n (%)			0.948
N0	129 (34.4%)	177 (47.2%)	
N1	30 (8%)	39 (10.4%)	
Residual tumor, n (%)			0.014
No	101 (24.1%)	172 (41.1%)	
Yes	73 (17.4%)	73 (17.4%)	

*IQR* interquartile range

### Mutation landscape, functional enrichment analysis, TME evaluation and tumor heterogeneity and stemness

As senescent cells with SASP can have a huge impact on the tumor immune microenvironment and have a dual role for the tumorigenesis and tumor progression [37, 38]. We furthermore evaluated the difference of mutation landscape between detrimental subtype and beneficial subtype and found the top ten genes were SPOP, FOXA1, KMT2C, APC, BSN, DNAH17, MYH6, EPPK1, ZNF536 and ZC3H13 with statistical significance (Fig. 3E). For functional analysis, DNA repair, MYC target, oxidative phosphorylation, proteasome and ribosome were highly enriched in detrimental SASP subtype (Fig. 3F, G). UV response DN, TGF-beta signaling, mitotic spindle, protein secretion, adherens junction, dorso ventral axis formation, arrhythmogenic right ventricular cardiomyopathy arvc and circadian rhythm mammal were enriched in beneficial SASP subtype (Fig. 3F, G).

**Fig. 4:**
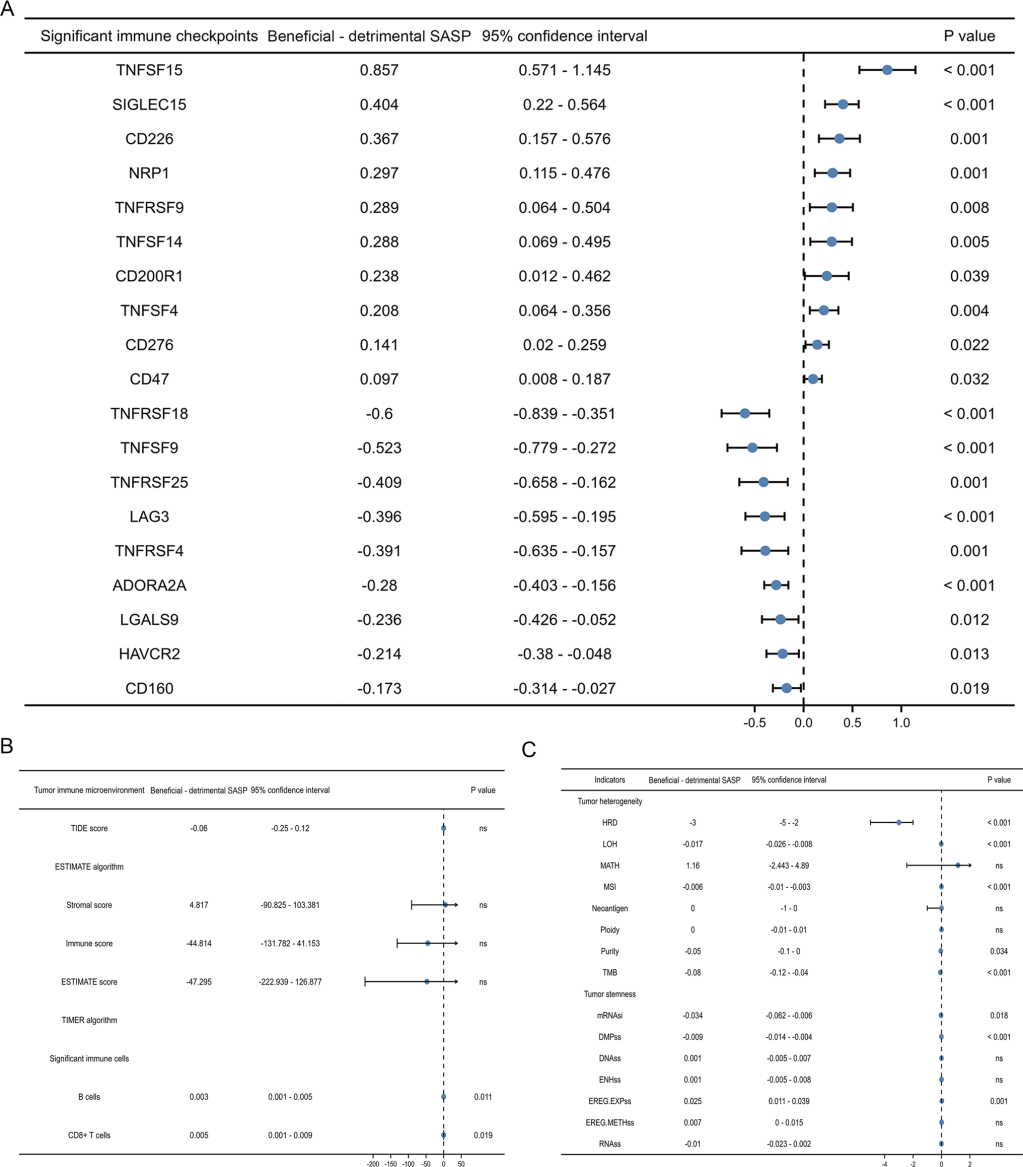
Tumor immune checkpoints and microenvironment, heterogeneity and stemness. **A** Forest plot showing the comparison of immune checkpoints between the two subtypes; **B** forest plot showing the comparison of tumor microenvironment assessment between the two subtypes; **C** forest plot showing the comparison of tumor heterogeneity and stemness between the two subtypes. *SASP* Senescence-associated secretory phenotype, *TIDE* tumor immune dysfunction and exclusion, *DMPss* differentially methylated probes-based stemness scores, *DNAss* DNA methylation-based stemness scores, *ENHss* enhancer elements/DNA methylation-based stemness scores; *EREG-METHss* epigenetically regulated DNA methylation-based stemness scores, *EREG.EXPss* epigenetically regulated RNA expression-based stemness scores, *RNAss* RNA expression-based stemness scores, *HRD* homologous recombination deficiency, *LOH* loss of heterozygosity, *NEO* neoantigen, *MATH* mutant-allele tumor heterogeneity, *TMB* tumor mutation burden, *MSI* microsatellite instability

In addition, we estimated the difference of significant immune checkpoints between detrimental SASP subtype and beneficial SASP subtype. The expression levels of TNFSF15, SIGLEC15, CD226, NRP1, TNFRSF9, TNFSF14, CD200R1, TNFSF4, CD276 and CD47 were significantly higher in beneficial SASP subtype and the expression levels of TNFRSF18, TNFSF9, TNFRSF25, LAG3, TNFRSF4, ADORA2A, LGALS9, HAVCR2 and CD160 were significantly higher in detrimental SASP subtype (Fig. 4A). For TME evaluation, we estimated the relative proportions immune cell types in PCa from TCGA cohort and found detrimental SASP subtype had significantly lower level of B cells and CD8+ T cells than beneficial SASP subtype (Fig. 4B). For tumor heterogeneity and stemness analysis, detrimental SASP subtype had significantly higher levels of HRD, LOH, MSI, tumor purity, TMB, mRNAsi and DMPss than beneficial SASP subtype but lower level of EREG.EXPss than beneficial SASP subtype (Fig. 4C).

## Discussion

PCa, a cancer associated with ageing that primarily affects males over 65, will unavoidably get a lot of attention as the world’s population ages [17, 57–59]. Cellular senescence, a key aspect of organismal ageing, is a crucial part of the ageing process. Evidence is mounting that cellular senescence and organismal ageing are linked in a complex web that both influences and contributes to one another [26, 60, 61]. Therefore, a thorough examination of the characteristics and mechanisms of cellular senescence as well as research into the connection between cellular senescence and the development and progression of PCa tumors are crucial for PCa treatment and prognosis prediction. Senescent cells usually contains two significant characteristics, one is the irreversible arrest of the cell cycle [62], which is associated with p53–p21–RB (retinoblastoma protein) pathway and p16^Ink4a^–RB pathway [63], and another is SASP. It has been found that SASP is closely related to the occurrence and development of various aging-related diseases, such as osteoarthritis, atherosclerosis and tumor [63]. In PCa, previous studies have shown that there were senescent cells accumulated in the aging prostate [41, 64]. Another study discovered that PCa cell development can be stopped by blocking the hydride transfer complex (HTC) in these cells and causing senescence in these cells [65]. We are aware of the significance of SASP in PCa thanks to these scientific data. Therefore, we identified three SASP-related genes (VGF, IGFBP3, and ANG), established a risk score, and uncovered detrimental and beneficial tumor subtypes based on these genes.

VGF is a neuroendocrine factor, which was first discovered in a pheochromocytoma cell line when exposed to nerve growth factor (NGF) [66]. VGF plays a crucial role in regulating metabolism and endoplasmic reticulum (ER) stress in both neurons and endocrine cells and it triggers pro-survival signaling pathways such as PI3K/AKT/mTOR and MAPK/ERK1/2 [67–71]. However, the role of VGF in the regulation of cancer cells is still not clear. A mouse in vitro study demonstrated that the production of VGF can cause the epithelial-mesenchymal transition (EMT), the dispersion of tumor cells, and resistance to EGFR inhibitors [72, 73]. Additionally, the survival of glioblastoma cells and tumour formation are linked to VGF expression in glioblastoma stem cells. In PCa, Michael et al. found VGF was involved in radioresistance of PCa cell lines [74] and Wenlin et al. found high expression of VGF was associated with low progression-free survival (PFS) [75]. Moreover, IGFBP3 is a member of the IGFBP family, which can bind to IGF-1 to limit its biological activity, and independently regulate cell growth and apoptosis [76, 77]. In tumor progression, IGFBP3 can either promote or inhibit tumor growth, but the specific mechanism is not fully understood yet. Through epidemiologic studies, high levels of IGFBP3 are associated with decreased risk of several common cancers, including PCa [78], breast cancer [79], colorectal cancer [80] and lung cancer [81]. A study reported that IGFBP3 might be involved in the early development of PCa through methylation [82]. These findings suggest that IGFBP3 plays an important role in the occurrence and development of PCa. ANG is a protein-encoding gene that mainly encodes angiogenin, which can interact with other angiogenic factors to regulate the process of angiogenesis. There are relatively few reports on the role and mechanism of ANG in the growth and invasion of PCa. A study found that as prostatic epithelial cells progressed from a benign to an invasive phenotype, the expression of angiogenin in prostatic tissue increased [83]. Meanwhile, studies using human PCa cell lines in athymic mice have demonstrated that blocking antibodies and antisense oligonucleotides targeted angiogenin can prevent the establishment, progression, and metastasis of PCa [84, 85]. In summary, based on these genes, we established beneficial SASP subtype and detrimental SASP subtype and constructed a risk score, which could accurately predict the prognosis of PCa patients. To better reveal the potential mechanisms of the two prognostic subtypes, we performed functional analysis, gene mutation, tumor heterogeneity and stemness, and TME assessment.

For functional enrichment analysis, we observed DNA repair and MYC target were highly enriched in detrimental SASP subtype. DNA repair genes play important roles in PCa. Men with hereditary mutations in either BRCA1 or BRCA2 had a greater risk of PCa [86]. Additionally, PCa patients who have germ cell mutations in their inherited germ line, notably BRCA2, tended to have worse clinical outcomes [87]. Additionally, it was discovered that genes involved in DNA repair have evolved genetic defects in about 23% of instances of metastatic PCa [88]. Therefore, the detrimental SASP subtype’s significant enrichment of DNA repair genes may be a factor in its poor prognosis. MYC is a type of significant oncogene that regulates gene transcription and promotes transformation [89]. Numerous studies have demonstrated a connection between MYC dysregulation and disruption of important biological processes, including cell cycle, tumor immune response, metabolism, cell competition, cell stemness, and a variety of other cellular activities [89–94]. The detrimental SASP subtype’s high enrichment of the MYC target correlates with greater biological process dysfunction and a worse prognosis.

In contrast, our study found TGF-beta signaling was enriched in beneficial SASP subtype. The TGF-beta is a pleiotropic cytokine that regulate multiple cellular functions, including regulation of embryonic growth and development, cell differentiation, proliferation and apoptosis, as well as secretion of extracellular matrix. TGF-beta has been demonstrated to have an inhibitory effect on PCa cells through encouraging the homeostatic regulation of apoptosis and proliferation in both normal and PCa prostate epithelial cells [95]. Besides, TGF-beta is crucial for regulating stromal-epithelial cell interactions as well as stromal cell activity [95]. Prostate epithelium would become cancerous if TGF-beta signalling in the stromal compartment were to be disrupted [96]. Interestingly, protein secretion was mainly enriched in beneficial SASP subtype and we speculated it may be related to the increasing androgen signaling. Hormone-refractory metastatic PCa showed a considerable decrease in androgen signalling and protein production when compared to hormone-naive metastatic PCa. This finding suggest that the decrease in androgen signaling and protein biosynthesis may contribute to the progression of PCa and increase the malignancy of tumor cells, therefore, the enrichment of protein secretion means more androgen signaling correlating to better prognosis [97].

Furthermore, we found the levels of B cells and CD8+ T cells were higher in beneficial SASP subtype. PCa is a kind of “cold” tumor, which means the level of immune cell is low in tumor tissues. However, the mechanism by which the level of immune cells infiltrating the TME is correlated with the prognosis of PCa patients remains unclear. Higher CD8+ T cell was associated with better survival after RP in the high-risk PCa cohort [98], which is consistent with our study. Another study found that compared with benign glands, the number of infiltrating B cells was significantly reduced in prostatic intraepithelial neoplasia and adenocarcinoma [99], which may imply that the malignancy of the tumor increase, the infiltrating B cells decrease.

In this study, we noticed that the level of HRD was significantly higher in detrimental SASP subtype. A crucial method for repairing DNA damage, homologous recombination repair (HRR) promotes genomic integrity and ensures the proper transmission of genetic information. It primarily occurs during the S and G2 phases of the cell cycle. By properly restoring the broken DNA sequence using a sister chromatid or homologous chromosome as a template, HRR is in charge of repairing double-strand breaks and other types of DNA damage [100]. This procedure is essential for reducing the buildup of chromosomal abnormalities and DNA mutations that can result in cancer and genetic diseases [100]. HRD occurs when DNA damage cannot be repaired by the homologous recombination repair process. Some known genes that encode homologous recombination proteins include BRCA1, BRCA2, ATM and so on [100–102]. In PCa, poly ADP-ribose polymerase inhibitor (PARPi) is currently a promising direction for the treatment of castration-resistant PCa [103]. In HRD tumor cells, an intact single-strand breaks (SSB) repair pathway is crucial for cell survival [104]. Synthetic lethality, which is caused when PARPi disrupt the SSB repair pathway and reduce PARP function, ultimately results in the death of tumor cells [104]. Detrimental SASP subtype with higher level of HRD means more sensitive to PARPi and may correlate with worse prognosis.

We also compared the differences of some significant immune checkpoints between beneficial and detrimental SASP subtype. Recent studies have provided increasing evidence that co-stimulatory signals transmitted via a subset of molecules belonging to the TNFR superfamily, which are critical for the development of protective immunity, as well as for the treatment of inflammatory and cancer immunotherapy and these molecules include OX40 (TNFRSF4), 4-1BB (TNFRSF9), CD27, DR3 (TNFRSF25), CD30 (TNFRSF8), GITR (TNFRSF18), TNFR2 (TNFRSF1B), and HVEM (TNFRSF14), which is possible to enhance the effectiveness of immunotherapeutic treatments for cancer [105]. However, our study found TNFRSF18, TNFSF9, TNFRSF25 and TNFRSF4 were expressed significantly higher in detrimental SASP subtype. We speculated this situation may be associated with tumor immune evasion. In detrimental SASP subtype, immune evasion occurred leading to a decrease in infiltrating CD8+ T cells, which in turn negatively feedbacked to upregulate these co-stimulatory molecules, which is associated with a worse prognosis.

Moreover, we have to admit the limitation that the expression of SASP-related genes does not mean that the corresponding proteins are secreted and contribute to senescent secretome. In fact, a large portion of the senescent secretome is made up of proteins that lack a secretion signal in their polypeptide sequence and are only secreted via microvesicles during the senescence of a particular cell.

## Conclusion

Based on three SASP-related genes, we developed two SASP-related prognostic subtypes and constructed a gene prognostic index, which were closely associated with BCR-free survival for PCa patients and might be important for the future research in the field of PCa.

## Data Availability

The results showed here are in whole or part based upon data generated by the TCGA Research Network: https://www.cancer.gov/tcga.
